# Impaired Autophagy of an Intracellular Pathogen Induced by a Crohn's Disease Associated ATG16L1 Variant

**DOI:** 10.1371/journal.pone.0003391

**Published:** 2008-10-13

**Authors:** Petric Kuballa, Alan Huett, John D. Rioux, Mark J. Daly, Ramnik J. Xavier

**Affiliations:** 1 Gastrointestinal Unit and Center for Computational and Integrative Biology, Massachusetts General Hospital, Harvard Medical School, Boston, Massachusetts, United States of America; 2 Program in Medical and Population Genetics, Broad Institute, Massachusetts Institute of Technology and Harvard University, Cambridge, Massachusetts, United States of America; 3 Université de Montréal, Montréal Heart Institute, Montréal, Québec, Canada; 4 Center for Human Genetics Research, Massachusetts General Hospital, Harvard Medical School, Boston, Massachusetts, United States of America; University of Cambridge, United Kingdom

## Abstract

The genetic risk factors predisposing individuals to the development of inflammatory bowel disease are beginning to be deciphered by genome-wide association studies. Surprisingly, these new data point towards a critical role of autophagy in the pathogenesis of Crohn's disease. A single common coding variant in the autophagy protein ATG16L1 predisposes individuals to the development of Crohn's disease: while ATG16L1 encoding threonine at amino acid position 300 (ATG16L1*300T) confers protection, ATG16L1 encoding for alanine instead of threonine (ATG16L1*300A, also known as T300A) mediates risk towards the development of Crohn's disease. Here we report that, in human epithelial cells, the Crohn's disease-associated ATG16L1 coding variant shows impairment in the capture of internalized *Salmonella* within autophagosomes. Thus, we propose that the association of ATG16L1*300A with increased risk of Crohn's disease is due to impaired bacterial handling and lowered rates of bacterial capture by autophagy.

## Introduction

Crohn's disease (CD) is a complex, multigenic disease with several identified genetic and non-genetic risk factors [1]–[4]. Despite this fundamental complexity, the known risk factors strongly support a role for the innate, adaptive immune response and autophagy in CD pathogenesis. Autophagy is a diverse process, with several forms described to date [5]. However, the most studied form is “macroautophagy” whereby cellular contents are encapsulated in double membraned autophagosomes and delivered to lysosomes, where they are degraded and their constituents recycled. This macroautophagic process is increased in times of cellular stress, such as starvation or growth factor withdrawal, for which the term “induced autophagy” has been suggested [6]. In addition to basal autophagy, where autophagosomes form constitutively in a stochastic fashion throughout the cytoplasm and encapsulate a non-selected cargo, there appear to be more selective forms of autophagy (typically induced by protein aggregates and bacterial pathogens) with cargo selection by as yet unknown signals [7], [8]. Autophagy is thought to protect the cell by eliminating or limiting the growth of bacterial pathogens, a process termed “xenophagy” [9]; therefore dysfunction of xenophagy might lead to persistent infection. In the case of *Salmonella enterica* subsp. *enterica* serotype Typhimurium (*S.* Typhimurium), it has been shown that xenophagy provides a significant contribution to pathogen control, and loss of autophagy results in enhanced bacterial replication within the intracellular niche [8]. Although the current evidence does not support autophagy as a universal defense against bacterial infection, the enhanced control of *S.* Typhimurium burden mediated by autophagy indicates that it forms part of the innate defense against *Salmonella* infection. Therefore, we shall refer to autophagy of internalized *Salmonellae* as anti-*Salmonella* autophagy.

Recently, a coding polymorphism in the autophagy gene ATG16L1 was identified as a risk factor for the development of CD [10], [11], and we demonstrated that ATG16L1 is essential for mammalian autophagy under a variety of induction stimuli, including *Salmonella* Typhimurium infection. In this study, we build upon our previous experiments, directly address the biochemical properties of ATG16L1 coding variants, and provide the first evidence that the ATG16L1*300A variant is associated with a loss-of-function phenotype upon challenge by an intracellular pathogen.

## Materials and Methods

### Antibodies

Mouse monoclonal antibodies used in this study were anti-FLAG (M2, Sigma, MO USA), anti-HA (16B12, Covance, NJ USA), Anti-GFP (B34, Covance) and anti-MYC (9E10, Covance). Anti-actin and anti-LC3B rabbit polyclonal antibodies were purchased from Sigma (MO, USA). Detection of beta Galactosidase protein was performed using a rabbit polyclonal antibody (Abcam, MA USA). In addition, two polyclonal antibodies against synthetic peptides corresponding to human ATG16L1 were purchased from Affinity BioReagents (CO USA) and were prepared by immunization of rabbits and affinity purified (sequences used for anti-ATG16L1-01 and anti-ATG16L1-02 were EKHDVPNRHEISPGHDGT and AEKAQEANRLNAENEKD, respectively).

### Plasmids and RNA interference (RNAi)

Human ATG16L1 isoforms 1 (NM_030803) and 2 (NM_017974) were amplified from cDNAs obtained from OriGene (MD USA), template cDNA for human ATG5 (NM_004849) was purchased from Open Biosystems (AL USA). Subsequent cloning of truncated versions of ATG16L1 was performed by polymerase chain reactions (PCR) using ATG16L1 isoform 1 as DNA template. ATG5 and ATG16L1 cDNAs were cloned into a modified version of pCMV (Clontech, CA USA) containing an N-terminal FLAG-, MYC- or HA-tag, as indicated. Expression plasmids coding for the ATG16L1*300A variant were generated from their *300T counterparts by site-directed mutagenesis. Further, site-directed mutagenesis was applied to introduce two silent mutations within the regions of ATG16L1-cDNA, whose corresponding mRNA regions are targeted by ATG16L1 small interfering RNAs (siRNAs) 1 and 2, respectively. ATG16L1 cDNAs resistant to siRNAs 1 or 2 were used to generate additional expression constructs by polymerase chain reaction and subsequently cloned into pCMV (untagged ATG16L1) or a modified version of pCMV carrying an in-frame C-terminal FLAG-tag. SiRNA duplexes were purchased from Invitrogen and have been previously validated [11], catalogue numbers for each duplex are: siRNA 1 – HSS147871; siRNA 2 – HSS147872.

To allow for stable suppression of ATG16L1 expression, a short hairpin RNA (shRNA) construct corresponding to ATG16L1 siRNA 1 was cloned into the unique restriction sites EcoRI and AgeI of pLKO.1. pLKO.1 cloning vector, pLKO.1 non-targeting control shRNA-vector, packaging plasmid pCMV-dR8.91 and envelope plasmid VSV-G/pMD2.G were purchased from the RNAi Consortium (The Broad Institute, MA USA).

The lentiviral construct, used to generate cell lines stably expressing GFP-hLC3B, was a kind gift from Dr. C. Münz (Rockefeller University, New York). The expression construct for beta-Galactosidase was kindly provided by Dr. M. Scheffner (University of Konstanz, Germany).

### Cell culture, transfections and viral infections

HeLa, HEK293, HEK293T and Caco2 cells were cultured in DMEM (Gibco, CA USA) supplemented with 10% iron-supplemented calf serum (CSFe) (Hyclone, UT USA) and 20 µg/ml gentamycin sulfate. Transfections of HeLa and HEK293 cells were performed using Lipofectamine 2000 (Invitrogen, CA USA), for transfections of HEK293T cells TransFectin (BioRad, CA USA) was used. Transfection of Caco2 cells was performed using an Amaxa Nucleofector system with Cell Line Nucleofector® Kit T, program B-24, according to the manufacturer's instructions (Amaxa, Germany). GFP-hLC3B- and shRNA-encoding lentiviral particles,were generated and transduced according to standard protocols given by the RNAi Consortium (The Broad Institute, MA USA) with minor modifications. For selection of Caco2 cells stably expressing control shRNA or shRNA directed against ATG16L1, respectively, puromycin (Sigma) was added to cell culture media at a final concentration of 6 µg ml^−1^.

### Bacterial-autophagy assays

Infections with *Salmonella enterica* sbsp *enterica* serotype Typhimurium strain SL1344 (*S.* Typhimurium) carrying a DsRed2 expression plasmid (Clontech) were performed as previously described [11]. The total number of bacteria per cell, and the number of GFP-LC3 positive bacteria were assessed in randomly chosen fields with at least 120 cells counted for each condition. The numbers of GFP-LC3 positive bacteria were then calculated as a percentage of total bacteria. Significance was assessed using the two-tailed, unequal variance Student's *t*-test. Representative images were taken using a Leica SP5 confocal microscope (Leica Microsystems Inc, IL USA) and are displayed as single optical sections from high-resolution z-stacks.

## Results

### Experimental design for coding variant-specific cell culture assays

**Figure 1:**
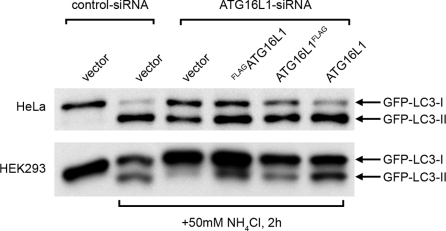
Differentially tagged ATG16L1*300T constructs show differential abilities to mediate basal autophagy. In a 12-well format, HeLa and HEK293 cells, stably transduced to express GFP-LC3, were co-transfected with control siRNA and empty FLAG-vector or ATG16L1 siRNA and empty FLAG-vector or expression constructs encoding ATG16L1 carrying no tag (‘ATG16L1’) a 3-fold FLAG-tag at its N-terminus (‘^FLAG^ATG16L1’) or C-terminus (‘ATG16L1^FLAG^’), as indicated. 48 h. post-transfection, 50 mM ammonium chloride (NH_4_Cl) was added for 2 h., where indicated, to block lysosomal turnover of GFP-LC3. Protein levels of GFP-LC3-I (cytosolic) and GFP-LC3-II (membrane-bound) were determined by Western blot analysis using anti-GFP-antibody.

We have previously demonstrated that human ATG16L1 is essential for autophagosome formation in human epithelial cells in the contexts of both serum withdrawal and bacterial infection [11]. However, the function(s) of ATG16L1 which are affected by the coding variation remain unknown. To this end, we applied a knock-down/reconstitution strategy, as previously described [12]. Within this system, expression of endogenous ATG16L1 is suppressed by means of RNAi, and ATG16L1 expression is reconstituted via transfection of an expression plasmid in which the ATG16L1 cDNA contains synonymous mismatches within the RNAi target sequence, preventing efficient RNAi targeting, but maintaining amino acid fidelity. Initially, we tested if and to what extent epitope tags might affect ATG16L1 expression constructs in their ability to rescue the loss of endogenous ATG16L1 expression. Therefore, we performed reconstitution assays (knock-down of endogenous ATG16L1 by RNAi and reconstitution of ATG16L1*300T expression by co-transfection of N-terminal tagged, C-terminal tagged and untagged, respectively, ATG16L1 expression plasmids) in cell lines stably expressing the autophagy marker GFP-LC3. Autophagy levels were determined via the extent of accumulation of membrane-bound GFP-LC3-II, serving as a positive marker of autophagosome formation [13], upon inhibition of lysosomal turnover in the absence of any further stimuli (basal autophagy). Surprisingly, both FLAG-tagged (N- and C-terminal, respectively) ATG16L1 expression constructs appear to be less competent than untagged ATG16L1*300T in their ability to rescue autophagy to levels seen with endogenous ATG16L1 (expression of control siRNA along with an empty expression vector) (Fig. 1). While the extent of reconstitution efficacy varies between HeLa cells and HEK293 cells, the trend seen for N-, C- and untagged ATG16L1 is consistent between both cell lines, suggesting that the placement of epitope tags has adverse effects upon the autophagy efficacy of ATG16L1 constructs. In summary, we are able to induce and functionally rescue loss of endogenous ATG16L1 expression using ATG16L1 RNAi-resistant expression constructs carrying no epitope tag.

### ATG16L1*300A is defective in mediating efficient anti-bacterial autophagy in HeLa and Caco2 cells

As the critical CD risk-conferring amino acid position 300 of ATG16L1 is within a region of mammalian ATG16L1 absent in yeast ATG16, we hypothesized that the specialized anti-bacterial from of autophagy would be affected rather than basal forms of autophagy, which rely on non-specific cargo selection. In addition, the high prevalence of the CD risk-conferring ATG16L1*300A variant in the human population most likely argues against a general deleterious autophagy phenotype. Thus, we reasoned that subtle differences in ATG16L1 variant function might be revealed in *Salmonella* infection assays [8], [11]. Therefore, we applied our knock-down/reconstitution system, including variant specific ATG16L1 reconstitution, followed by infection with *S.* Typhimurium infection and measured the percentage of GFP-LC3 captured bacteria.

**Figure 2:**
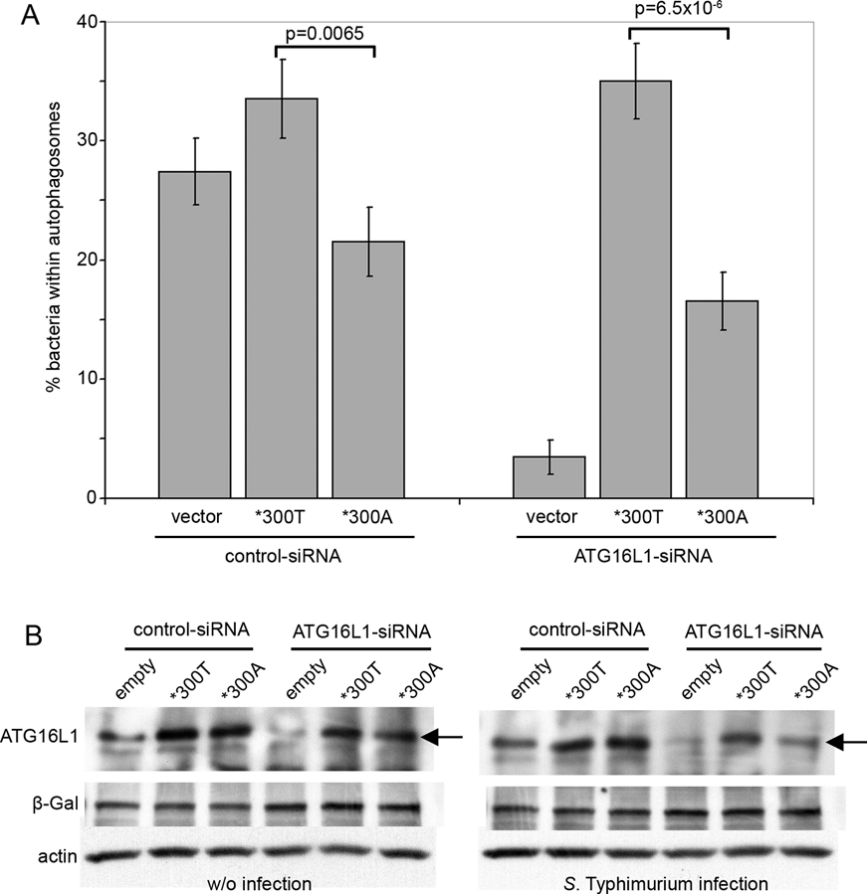
ATG16L1*300T is defective in mediating anti-*Salmonella* autophagy in HeLa cells. (A) HeLa cells stably expressing GFP-LC3 were co-transfected with control or ATG16L1-targeting siRNA and empty vector, ATG16L1*300T or ATG16L1*300A expression constructs, as indicated. 48 h. following transfection, cells were infected with *S.* Typhimurium (SL1344) for 1 h. and fixed. The percentage of internalized bacteria captured within GFP-LC3-positive autophagosomes was calculated and is shown as means with error bars representing standard errors. P values were calculated as described in Material and Methods. (B) HeLa cells were co-transfected as described in Fig. 2A with the additional co-transfection of a beta-galactosidase expression plasmid in each sample. 48 h. following transfection, one set of cells was left untreated (w/o infection) while the other set was infected with *S.* Typhimurium (SL1344) for 1 h. 5% of each lysate were used for straight Western blotting and detection of beta-galactosidase (β-Gal) (indicator of transfection efficiency) and actin (loading control). ATG16L1 expression levels were detected in the remaining 95% of each lysate by immunoprecipitation with anti-ATG16L1-02 antibody and Western blot analysis using anti-ATG16L1-01 antibody. Note the loss of ATG16L1*300A protein (compared to *300T and without infection) during *S.* Typhimurium infection – arrowed. w/o, without.

In the presence of endogenous ATG16L1, ATG16L1*300A-expressing HeLa cells are consistently less able to capture bacteria within autophagosomes than ATG16L1*300T expressing cells (Fig. 2A, left panel). Strikingly, in the absence of endogenous ATG16L1, ATG16L1*300A-expressing cells are markedly less effective in anti-*Salmonella* autophagy, recovering less than half of the activity displayed by the ATG16L1*300T protein (Fig. 2A, right panel). In addition, in the absence of endogenous ATG16L1 protein, levels of ATG16L1*300A were consistently lower than ATG16L1*300T after bacterial challenge (data not shown and Fig. 2B). This effect was not seen without infection and was only observed in the absence of endogenous ATG16L1 (Fig. 2B). This suggests that the relative instability of the *300A variant protein can be largely compensated for by endogenous levels of ATG16L1*300T, or perhaps suggesting that ATG16L1*300A is only targeted within a functional ATG5/ATG12/ATG16L1 complex while over-expressed ATG16L1 only inefficiently incorporates into pre-existing ATG5/ATG12/ATG16L1 complexes as suggested by Fujita et al [14].

**Figure 3:**
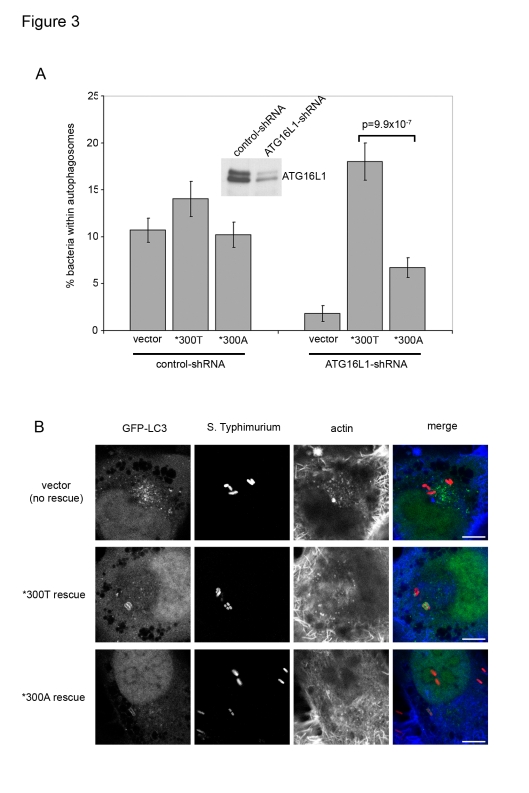
In the human gut epithelial cell line Caco2, ATG16L1*300A is impaired in its ability to mediate anti-*Salmonella* autophagy. (A) Caco2 cells were transduced with lentiviruses expressing non-targeting shRNA (control-shRNA) or ATG16L1-targeting shRNA (ATG16L1-shRNA). Protein levels of endogenous ATG16L1 were detected in stably transduced cells by immunoprecipitation with anti-ATG16L1-02 antibody and Western blot analysis using anti-ATG16L1-01 antibody (inset). The generated CaCo2 cell lines were nucleofected with both GFP-LC3 and either control (vector), ATG16L1*300T or *300A expression constructs, as indicated. 48 h. after nucleofection cells were infected with *S.* Typhimurium for one hour and fixed. The percentage of GFP-LC3-positive bacteria and P values were determined as described in Fig. 2A. (B) Representative confocal optical sections of infected ATG16L1-deficient and ATG16L1 allele-specific resconstituted Caco2 cells. GFP-LC3 (green in merge), SL1344 (red in merge) and actin (blue in merge) are shown, scale bars represent 10 µm. Note the lack of GFP-LC3-marked membranes around internalized bacteria in knock-down cells (top row), recovery of autophagy in ATG16L1*300T-reconstituted cells (middle row) and partial recovery in *300A-reconstituted cells (bottom row).

We confirmed these findings in a human gut epithelial cell line by establishing a modified knock-down/reconstitution strategy in CaCo2 cells (Fig. 3A). These cells were lentivirally transduced to generate a stable knock-down of ATG16L1 protein expression (Fig. 3A inset) and subsequently nucleofected with ATG16L1 reconstitution constructs and GFP-LC3. The phenotype observed in Caco2 cells closely mirrored that of our HeLa system. Under conditions of endogenous ATG16L1 ablation, expression of ATG16L1*300A failed to fully rescue autophagy of *S.* Typhimurium, whereas ATG16L1*300T yielded robust anti-*Salmonella* autophagy. Representative images of infected Caco2 cells showing that cells expressing either ATG16L1 variant are able to capture *Salmonella* within autophagosomes, albeit at a reduced rate in the case of ATG16L1*300A, are shown in Figure 3B.

Taken together, these findings demonstrate that the CD-associated variant ATG16L1*300A is less able to mediate anti-*Salmonella* autophagy. We proposed that the underlying mechanism might be related to an impaired ability of the *300A variant to interact with other proteins within the autophagic complex.

### The ATG16L1*300A variant exhibits no defect in dimerisation or binding to ATG5

**Figure 4:**
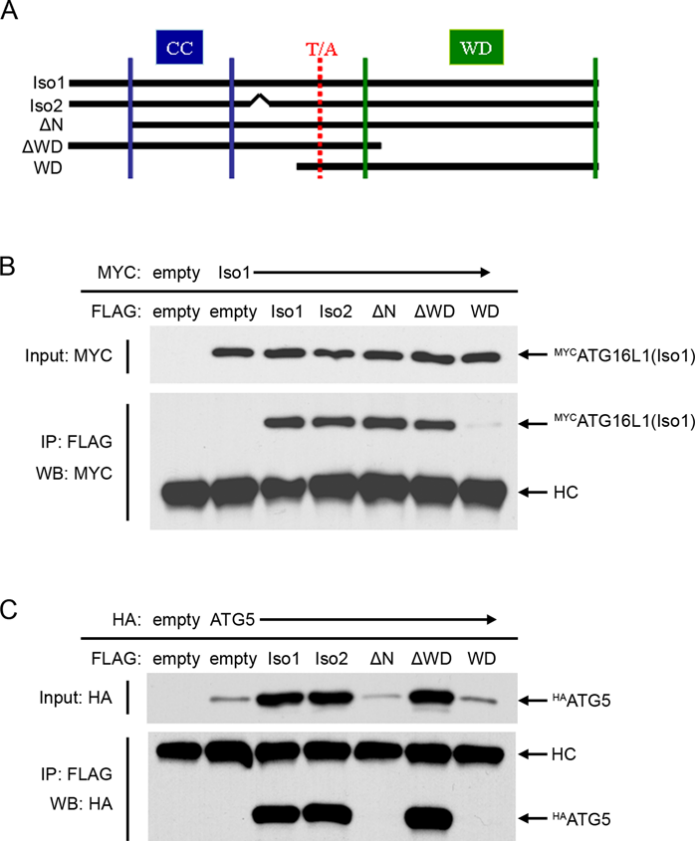
Human ATG16L1 forms homodimers depending on a region containing the coiled coil domain and heterodimers with human ATG5 via its very N-terminus. (A) Schematic view of ATG16L1 expression constructs used in this manuscript. Amongst a number of predicted isoforms, isoform 1 (Iso1) and isoform 2 (Iso2) of human ATG16L1 reflect homologues of mouse Apg16L isoforms β and α, respectively, and are the most prominently known isoforms in humans. ATG16L1 Iso1 represents the longest human isoform and encodes for 607 amino acids with a predicted coiled coil domain (amino acids 78–230, CC) and seven WD domains (amino acids 320–359, 364–403, 406–445, 447–484, 486–525, 532–573, 575–607, respectively, WD). Amino acid position 300 can be encoded by threonine or alanine (T/A), and individuals carrying alleles coding for alanine (ATG16L1*300A) show a significantly increased risk for the development of CD. Iso2 lacks a stretch of 19 amino acids (amino acids 266–284 of Iso1) resulting in a shorter 588 amino acid protein. For mutational studies, three truncated expression constructs of Iso1 have been generated encoding amino acids 85–607 (ΔN), amino acids 1–341 (ΔWD) and amino acids 286–607 (WD), respectively. (B and C) HEK293T cells were co-transfected with FLAG-tagged expression constructs of ATG16L1 (as shown in Fig. 4A) and MYC-tagged ATG16L1 Isoform 1 (B) or HA-tagged ATG5 (C), respectively. 40 h. post-transfection cells were harvested and lysed. While 10% of each lysate served as input-control, the remaining lysate was incubated with goat anti-mouse beads and anti-FLAG-antibody overnight at 4°C to immunoprecipitate (IP) FLAG-ATG16L1 constructs. Over-expressed (input) and co-precipitated MYC-tagged ATG16L1 Isoform 1 were determined by Western blotting using an anti-MYC antibody (Fig. 4B); ATG5 was detected using anti-HA-antibody (Fig. 4C). Please note that under the conditions used only the monomeric (not conjugated to ATG12) form of ATG5 was expressed in significant amounts, in addition ATG5 protein levels appear elevated/stabilized upon binding to ATG16L1. HC: heavy chain. Empty: vector encoding the corresponding tag only.

Since yeast and murine ATG16(L1) proteins have the capacity to build homodimers mediated by their coiled-coil domain and are able to interact with ATG5 depending on their very N-terminus [15]–[17], it was tempting to speculate that corresponding domains of human ATG16L1 will act in the same manner within the human autophagy system. To address this issue, in addition to our ATG16L1 isoform 1 constructs (ATG16L1*300T and *300A), we constructed a panel of expression constructs reflecting ATG16L1 isoform 2, and three truncated forms of ATG16L1*300T (Fig. 4A). The ATG16L1 isoform 2 lacks a single exon of unknown function between the coiled-coil domain and the WD repeats. The construct ΔN lacks the N-terminal domain, but retains all other features of isoform 1. Since the WD repeat region is entirely absent from the yeast ATG16 homologue, two synthetic truncations were made, either lacking all WD repeats (ΔWD), or consisting of solely the WD repeats, plus a small undefined region distal to the coiled-coil (WD).

We investigated which of these constructs were able to co-precipitate human ATG16L1 isoform 1 and human ATG5 using over-expression of N-terminal tagged constructs in HEK293T cells followed by co-immunoprecipitation assays. We used tagged constructs, since this was the only way to immunoprecipitate all truncated versions and to distinguish ATG16L1*300T and *300A. In addition, recent data demonstrate that an inhibitory effect of N-terminal tagged ATG16L1 can be overcome by over-expression of ATG5 and ATG12 [14]. This suggests that excess levels of ATG16L1 are the cause of inhibition, but the biochemical properties of ATG16L1 are not affected. We therefore propose that N-terminal tagged ATG16L1 expression constructs are suitable for biochemical studies such as co-immunoprecipitation assays, yet functional assays are likely to be more reliable using ATG16L1 constructs carrying no epitope tag.

First, MYC-tagged ATG16L1*300T-isoform 1 or HA-tagged ATG5 were co-expressed with FLAG-tagged versions of each of the described ATG16L1*300T constructs. Immunoprecipitation was performed using anti-FLAG antibody and co-precipitated proteins were detected by Western blotting using a MYC-specific antibody (for ATG16L1 isoform 1) or HA-specific antibody (for ATG5).

Using this technique, we were able to demonstrate that human ATG16L1 requires a region between amino acids 85 through 285 to interact with other ATG16L1 moieties, most likely reflecting self-dimerisation mediated by the coiled-coil domain (Fig. 4B). Notably, the WD domains of human ATG16L1, which are absent in yeast ATG16, failed to co-immunoprecipitate ATG16L1. In addition, ATG16L1 interacts with ATG5 via the N-terminal domain of ATG16L1 (Fig. 4C), as no binding of ATG5 was observed for any of the ATG16L1 constructs missing the N-terminal 84 amino acids. Additionally, in the absence of exogenous ATG16L1 binding, the level of ATG5 protein was markedly reduced. Therefore, we suggest that the ATG5/ATG16L1 interaction influences the turnover rate of ATG5 and may serve to regulate the rate of autophagy.

Next, we examined whether ATG16L1*300T and the coding variant *300A differ in their capacities to form ATG16L1 homodimers and to bind and stabilize monomeric ATG5. FLAG-tagged ATG16L1*300T or *300A were co-expressed with HA-tagged ATG5 or MYC-tagged ATG16L1*300T or *300A. Co-precipitation assays were performed as before, using FLAG antibody for immunoprecipitation followed by Western blotting with MYC- or HA-antibody to detect co-precipitated proteins.

**Figure 5:**
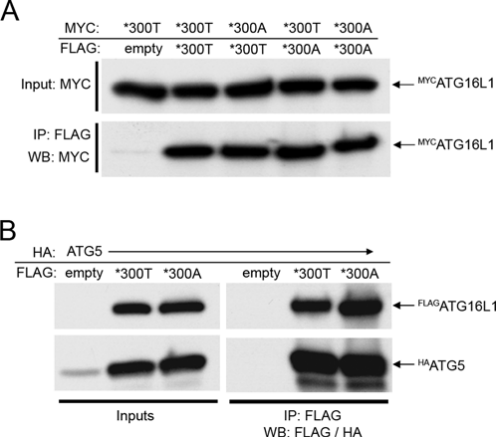
The formation of ATG16L1-homodimers and ATG16L1-ATG5-heterodimers is not affected by the *300A/T polymorphism. (A) HEK293T cells were co-transfected with FLAG and MYC-tagged ATG16L1*300A or *300T, as indicated. FLAG-tagged proteins were immunoprecipitated using anti-FLAG antibody. Both the inputs of MYC-ATG16L1 and co-precipitated MYC-ATG16L1 are shown. (B) HEK293T cells were co-transfected with FLAG-tagged ATG16L1*300A or *300T and HA-tagged ATG5, respectively, as indicated. Shown are inputs, as well as immunoprecipitated FLAG-ATG16L1 and co-precipitated monomeric HA-ATG5. Under the conditions used, only the monomeric (not conjugated to ATG12) form of ATG5 was expressed in significant amounts.

In these co-immunoprecipitation assays, we were not able to detect any significant difference between ATG16L1*300T and *300A with regard to ATG16L1 dimerisation, ATG5 binding or the stabilization of monomeric ATG5 (Fig. 5A and B). These observations argue against a defect in classical autophagy mediated by the ATG16L1*300A variant, since the key features of ATG16L1 (binding of ATG5 and other ATG16L1 moieties) needed to form the ATG16L1/ATG5/ATG12 complex are not affected by the ATG16L1*300A variant.

### The ATG16L1*300T and *300A coding variants are both fully competent in mediating basal autophagy

**Figure 6:**
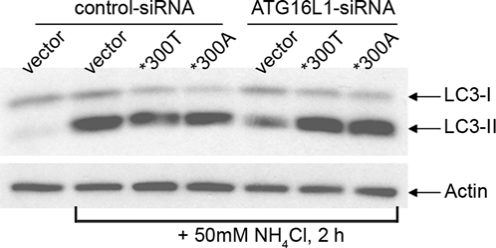
Overall levels of basal autophagy are not affected by the ATG16L1*300A polymorphism. HeLa cells were transfected as described in Fig. 2A. 48 h. post-transfection 50 mM ammonium chloride (NH_4_Cl) was added for 2 h. where indicated. Protein levels of LC3 and actin were detected by Western blotting using anti-LC3B and anti-actin antibodies, respectively.

To test whether ATG16L1*300A might affect the overall rate of basal autophagy, we applied our functional knock-down/reconstitution system using untagged ATG16L1*300T and *300A, respectively. We co-transfected HeLa cells with control siRNA or siRNA directed against ATG16L1 and either empty vector or ATG16L1 variants, followed by treatment with ammonium chloride to block turnover of the autophagy marker LC3 in autolysosomes. Strikingly, expression of either ATG16L1 variant was sufficient to reconstitute autophagosome formation in the absence of endogenous ATG16L1 (Fig. 6). These findings indicate that the ATG16L1*300A variant is unaffected in its ability to mediate basal autophagy. In addition, the recently reported inhibition of autophagy seen upon over-expression of N-terminal tagged ATG16L1 in the presence of endogenous ATG16L1 does not seem to be shared by untagged ATG16L1 constructs (Fig. 6), supporting accuracy of data observed within our experimental assay conditions.

## Discussion

The strong association with CD of two autophagy-associated genes (ATG16L1 and IRGM), both with known roles in anti-bacterial autophagy, is strong evidence for a causal role for altered autophagy in the pathogenesis of CD. Given the essential role for autophagy in development and metabolism, it is difficult to conceive that CD-associated mutations would have marked systemic effects upon overall levels of autophagy. Our understanding of autophagy has been largely informed by the characterization of the yeast system, although increasing interest in mammalian autophagy has driven a series of recent discoveries [6], [18], [19]. Despite their common essential role for autophagy in yeast and mammalian cells [11], [17], ATG16(L1) proteins from yeast and mammals show notable differences. Mammalian ATG16L1 proteins share the core domains of the yeast ATG16 molecule, but possess additional WD-repeat domains [10], [16], [20]. Therefore, we hypothesized that the WD-repeats of ATG16L1 display additional protein-protein interactions over the yeast molecule, and these interactions may be necessary for specialized forms of autophagy, such as anti-bacterial autophagy. The positioning of the CD-associated coding SNP (ATG16L1*300A or T300A) close to the WD allows the possibility that the CD-associated variant exhibits a phenotype in anti-bacterial autophagy. In addition, the role of the IRGM protein appears to be most prominent in anti-bacterial autophagy, further suggesting that anti-bacterial autophagy contributes to the pathogenesis of CD.

In order to investigate the role of ATG16L1 variants in autophagy, we applied a knock-down and reconstitution system using RNAi to ablate endogenous ATG16L1, while reconstituting ATG16L1 expression with RNAi-resistant cDNA constructs. We suggest that this approach is likely to reveal subtle and intermediate phenotypes likely to be characteristic of commonly-carried genetic variants. Additionally, in accordance with observations for murine ATG16L1 [14], we observed an inhibitory, or at least functionally compromised, effect mediated by N-terminal tagged human ATG16L1. While we believe that this is in part due to an abnormally high stability of N-terminal tagged proteins (P. Kuballa, unpublished data), we have been able to successfully circumvent inhibition by using ATG16L1 constructs carrying no tag, regardless of the exact mechanism of inhibition/dysfunction related to N-terminal tagged ATG16L1.

Using this system, we were able to demonstrate that basal levels of autophagy are unaffected by the presence of the CD-associated ATG16L1 mutation. Indeed, the interactions of ATG16L1 with other ATG16L1 moieties as well with ATG5 appear to be unaffected by the ATG16L1*300A/T polymorphism. This is perhaps not entirely surprising, since we were able to demonstrate that regions of ATG16L1 essential for interactions with ATG5 and other ATG16L1 moieties lie upstream of amino acid 300, which is affected by the identified functional SNP.

However, we were able to observe a marked decrease in the efficiency of anti-bacterial autophagy in both HeLa and CaCo2 cells expressing the CD-associated ATG16L1*300A allele compared to cells expressing the ATG16L1*300T allele, suggesting that handling of intracellular pathogens or internalized antigens might be altered in patients possessing the ATG16L1*300A allele. In addition, impairment of anti-bacterial autophagy correlated with a decrease in ATG16L1*300A expression levels in HeLa cells, while under the conditions used for functional assays (nucleofection of 5×10^5^ cells) expression levels of ATG16L1 were below the detection limit in the number of Caco2 cells, which survived the nucleofection procedure (Please note, that expression levels shown in Fig. 3A, inset have been derived from 10 cm plates). Thus, we are unable to definitively state whether the decrease in ATG16L1*300A protein stability reflects the mechanism of impairment, or if deficient or excessive protein-protein interactions of *300A compared to *300T are responsible for impaired capture of bacteria within autophagosomes.

However, whilst *Salmonellae* are not the causative agents of CD, nor present in the majority of patients, we anticipate that many of the cellular autophagy mechanisms necessary for microbe recognition and processing are shared for different intracellular pathogens. Thus, we believe that patients with the ATG16L1*300A allele are likely to be fully competent for the homeostatic functions of autophagy, yet may exhibit altered responses to bacterial components and infection in the gut milieu, where antigen burden is high. Whilst one advantage of the cell culture system used in this study is reflected by a direct comparison of ATG16L1 coding variants within an otherwise identical genomic background, we are currently seeking to confirm our results in cells derived from patients carrying the ATG16L1*300T or ATG16L1*300A alleles. However, a critical control, rescue of phenotypes in ATG16L1*300A-expressing cells from patients by exogenous expression of ATG16L1*300T, is likely to be challenging due to technical limitations.

Since autophagy is involved in the cross-presentation of intracellular antigens via MHC class II, it will be of interest to observe whether antigen presentation is modified in the context of ATG16L1*300A compared to ATG16L1*300T expressing cells [21]. This may be of particular importance in the gut microenvironment, where continual sampling, presentation and response to luminal antigens are performed by M cells, Paneth cells and dendrites extending between epithelial cells. Our observation that the ATG16L1*300A protein may exhibit altered stability under conditions of high microbial load also suggests that defects are most likely to become functionally relevant under such conditions. In addition, it is possible that modifications of autophagy may alter the host adaptive responses to self antigens via changes in cross-presentation of endogenous peptides [21].

Together, our observations suggest that the net effect of ATG16L1 coding variation will depend upon the balance between the high microbial load in the gut and the ability of autophagy to mediate defense against invading pathogens and internalized self- and non-self antigens.
